# Hepatic and abdominal adiposity in type 2 diabetes as assessed with machine learning on computed tomography scans

**DOI:** 10.1111/dom.70557

**Published:** 2026-02-18

**Authors:** Richard H. Tran, Pavan Raghupathy, Mohamad Hazim, Elizabeth Thompson, Sophia Swago, Abhijit Bhattaru, Matthew MacLean, Jeffrey T. Duda, James Gee, Charles Kahn, Daniel J. Rader, Arijitt Borthakur, Walter R. Witschey, Hersh Sagreiya

**Affiliations:** ^1^ Department of Radiology, Perelman School of Medicine University of Pennsylvania Philadelphia Pennsylvania USA; ^2^ Department of Medicine University of Massachusetts Chan Medical School Worcester Massachusetts USA; ^3^ Department of Genetics, Perelman School of Medicine University of Pennsylvania Philadelphia Pennsylvania USA; ^4^ Leonard Davis Institute of Health Economics University of Pennsylvania Philadelphia Pennsylvania USA

**Keywords:** abdominal adiposity, artificial intelligence, computed tomography, hepatic steatosis, machine learning, subcutaneous adipose tissue, type 2 diabetes, visceral adipose tissue

## Abstract

**Aims:**

The combined assessment of multiple abdominal imaging traits in relation to type 2 diabetes remains incompletely characterised. The study examines these relationships on computed tomography (CT) scans from a large‐scale, racially diverse, disease‐focused medical biobank.

**Materials and Methods:**

Deep learning algorithms were applied to patients with abdominal CT scans in the Penn Medicine BioBank to quantify image‐derived phenotypes, including spleen‐hepatic attenuation difference (SHAD) for hepatic steatosis (HS), liver and spleen volumes (SV), abdominal visceral and subcutaneous adipose tissue (VAT and SAT, respectively) and visceral‐to‐subcutaneous ratio (VSR). One thousand five hundred and ninety‐four patients (62 years, 49.4% male, 59.3% White), comprising 950 nondiabetics and 644 diabetics, were included in analysis with diabetes status determined by a 6.5% haemoglobin A1c cutoff.

**Results:**

Diabetic patients had greater HS (SHAD −4.49 vs. −6.88 Hounsfield units, *p* = 1.34 × 10^−8^), steatosis prevalence (41.8% vs. 27.7%, *p* = 4.85 × 10^−9^) and VSR (0.62 vs. 0.55, *p* = 1.69 × 10^−3^) than nondiabetics. In multivariate analyses adjusting for age, sex, race and body mass index (BMI), diabetes was independently associated with SHAD (odds ratios [OR] 1.04, 95% confidence interval [1.02–1.05]), SV (OR 4.53 [1.89–10.99]) and VSR (OR 2.87, [1.96–4.20]). Combined regression analysis showed no relationship between splenomegaly and type 2 diabetes once controlling for hepatic factors (OR 1.08, [0.95–1.23]), but uncovered a stronger VSR correlation (OR 1.40, [1.20–1.63]) than BMI (OR 1.14, [1.01–1.29]).

**Conclusions:**

Hepatic steatosis, hepatomegaly and visceral adiposity on CT are associated with type 2 diabetes. Hepatic changes may influence spleen size effects on diabetes. VSR can serve as an alternative to traditional obesity metrics to accurately reflect diabetes risk.

## 1 INTRODUCTION

Diabetes mellitus is projected to impact over 780 million people globally by 2045.^1^, ^2^ Despite its prevalence, the disease is typically underdiagnosed and detected at later stages, leading to missed opportunities for early intervention that can prevent serious associated cardiometabolic diseases.^3^, ^4^, ^5^ Imaging represents a potential opportunistic screening tool, whereby information obtained during routine or unrelated clinical assessments can be leveraged to determine the presence of type 2 diabetes and its associated risk factors.^6^

With over 85 million computed tomography (CT) scans performed in the United States each year, there is opportunity to harness such data to identify patients with type 2 diabetes.^7^ The advancement of machine learning in radiology has allowed for efficient and rapid analysis of substantial amounts of data, with applications seen in the assessment of stroke, osteoporosis and sarcopenia on CT scans.^8^, ^9^, ^10^, ^11^ These approaches allow for extraction and calculations of quantitative imaging measurements, or image‐derived phenotypes (IDPs), from radiological scans to explore novel disease associations, expanding our ability to perform phenotypic assessments as part of clinical care and improve clinical decision making.^11^, ^12^, ^13^ As IDPs are automatically quantified and established at the time of the study, their use presents no additional risk to the patient and provides an opportunity to detect findings that could be missed by radiologists, especially given the large volume of studies they typically read.

Prior studies have independently quantified abdominal IDPs, including HS through the spleen‐hepatic attenuation difference (SHAD), central adiposity which includes visceral adipose tissue (VAT) located between organs and subcutaneous adipose tissue (SAT) located peripherally under the skin, liver organ size, as well as evaluated their relationship with type 2 diabetes on CT scans.^13^, ^14^, ^15^, ^16^, ^17^, ^18^ However, an integrated assessment of these abdominal imaging phenotypes remains incompletely characterised. Moreover, there has been limited attention to spleen size and its relationship to type 2 diabetes, even though splenic metrics are routinely used to calculate HS.^14^, ^16^, ^19^, ^20^ A comprehensive approach may clarify interrelationships among these abdominal IDP values and improve type 2 diabetes risk stratification beyond individual parameters. CT scans are particularly well‐suited for such large‐scale cumulative analyses as alternative non‐invasive diagnostic methods such as magnetic resonance imaging are expensive and not as cost‐effective, while ultrasound demonstrates inter‐reader variability in qualitative steatosis assessment.^21^, ^22^, ^23^, ^24^

In this study, we leveraged body composition analysis to uncover imaging markers of metabolic dysfunction in the diabetic population. We utilised electronic health record (EHR) data from participants enrolled in the Penn Medicine BioBank (PMBB) to identify patients with non‐contrast abdominal CT scans and an associated haemoglobin A1c (HbA1c) value of ≥6.5% as a diabetes diagnosis. Using machine learning methods, we automated the analysis of certain abdominal IDPs, including SHAD, liver volume (LV), spleen volume (SV), abdominal VAT, abdominal SAT and visceral‐to‐subcutaneous fat ratio (VSR). We aim to quantify the differences in these IDPs between individuals with and without type 2 diabetes, as well as explore the combined associations of HS, abdominal fat distribution and hepatosplenic organ size with the presence of type 2 diabetes.

## 2 MATERIALS AND METHODS

### 2.1 Institution

This study utilised data from the PMBB, a biomedical database consisting of advanced imaging, biological samples and other EHR data from over 250 000 patients within the University of Pennsylvania Health System, a multi‐hospital network headquartered in Philadelphia, PA. All enrolled patients provided informed consent for researchers to access their EHR.

### 2.2 Study cohort imaging data

**FIGURE 1:**
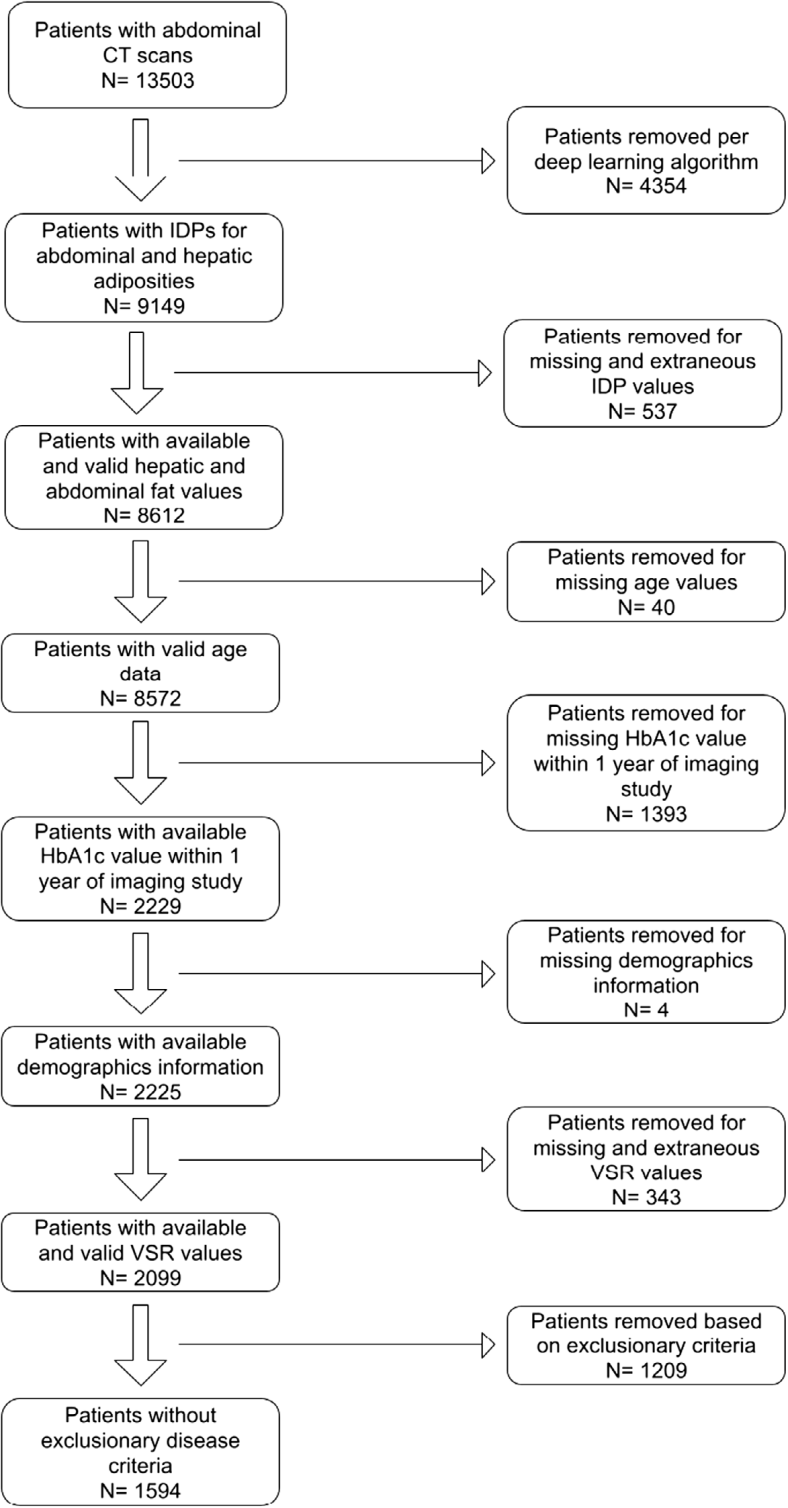
Patient flowchart. Number of patients in Penn Medicine BioBank with available quantified image derived phenotypes related to hepatic and abdominal fat. Patients with applicable phecodes (alcohol use disorder, hepatitis and end‐stage liver disease) were excluded from the final analysis. CT, computed tomography; HbA1c, haemoglobin A1c; IDPs, image‐derived phenotypes; VSR, visceral‐to‐subcutaneous ratio.

From 1998 to 2019, 13 503 patients were identified that received abdominal and pelvic CT scans designated by the appropriate Current Procedural Terminology codes (Figure S1). Nine thousand one hundred and forty‐nine PMBB patients had abdominal CT scans that yielded IDP values for hepatic and abdominal adiposity based on our deep learning algorithm (discussed below). To associate a single SHAD value to each patient, we first selected the image series within each CT examination that had the SHAD value closest to the median SHAD of that entire exam. Of note, a patient can have multiple CT exams on different dates, and each CT exam has multiple series. Then, we grouped the data by PMBB identification number, which identified each unique patient, and selected the study that had the greatest SHAD value per patient. This was done in order to ensure the maximum degree of hepatic steatosis was captured for each patient. Patients with SHAD values within −30 Hounsfield units (HU) and 30 HU were included. Certain exclusion criteria were applied sequentially to obtain the patient population of interest (Figure 1). In brief, missing and extraneous IDPs were removed. Patients with missing age values were removed. We used mapped phecodes, which are disease phenotypes derived from the International Classification of Diseases, Ninth Revision (ICD‐9) (https://www.phewascatalog.org/phecodes) to apply exclusionary criteria for certain diseases (Figure S2).^25^ Additionally, those without HbA1c values within a year of the CT study date were excluded from the analysis. For patients with multiple HbA1c measurements within the 1‐year window, the value closest in time to the CT study was used. Patients with missing demographic information (race, body mass index [BMI] and sex) were excluded. VSR was calculated by dividing VAT by SAT. Patients without VSR or outlier values were excluded. Patients with alcohol use disorder, alcohol‐related liver disease, end‐stage liver disease and viral hepatitis phecodes were excluded to remove potential confounders. The final cohort comprised 1594 patients and data were collected for age, sex, race/ethnicity, BMI, HbA1c and medication use (Figure 1). Type 2 diabetes status was defined based on HbA1c values ≥6.5% obtained within 1 year of the imaging study. Race and ethnicity were included in the analysis to account for population‐level differences in BMI cutoffs for obesity.^26^, ^27^ Patients with their ethnicity classified as Hispanic or Latino were considered as another race cohort, independent of race. Medication use was summarised descriptively to characterise the cohort.

### 2.3 Machine learning algorithm for adipose quantification

**FIGURE 2:**
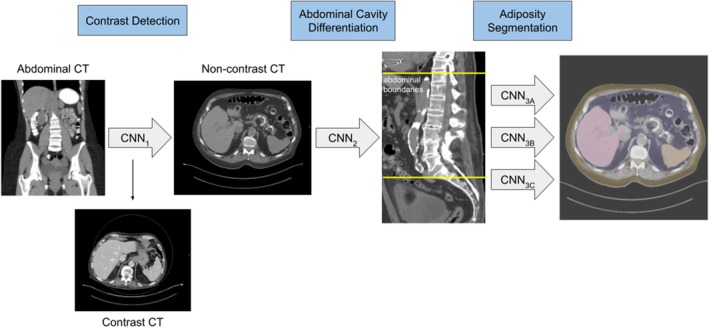
Deep learning method for the segmentation and quantification of hepatic and abdominal adiposity. CNN_1_ identified non‐contrast from contrast computed tomography (CT) scans. CNN_2_ delineated the boundaries of the abdominal compartment. The third convolutional neural network (CNN) simultaneously segmented the liver (CNN_3A_) (in pink), spleen (CNN_3B_) (in orange), and characterised visceral adipose tissue area (in purple) from subcutaneous adipose tissue area (in yellow) (CNN_3C_). Hepatic steatosis was diagnosed if spleen‐hepatic attenuation difference was greater than −1 Hounsfield unit (HU) or liver mean attenuation was less than 40 HU.

A well‐defined deep learning method that our group previously developed (Dice Score coefficients ranging from 0.92 to 1.00) was applied for the segmentation and quantification of liver fat, liver and splenic volume and abdominal adipose tissue.^13^, ^15^, ^28^, ^29^ In short, axial abdominal CT scans that had high‐pass filter kernel resolutions or those with image slice thickness <2 mm were excluded to eliminate noise. Imaging studies with <10 slices were excluded to avoid incomplete studies. The applied deep learning method consists of three convolutional neural networks (CNNs) (Figure 2): CNN_1_ identified non‐contrast CTs, as contrast bolus affects peak splenic and hepatic attenuation in a time‐dependent manner.^30^ CNN_2_ defined certain axial slices as the abdominal compartment boundaries between the inferior thoracic cavity and L5 vertebrae. Lastly, fully automated segmentation of the liver (CNN_3A_), spleen (CNN_3B_) and delineation of abdominal SAT and VAT were conducted (CNN_3C_). Liver, spleen, and abdominal SAT and VAT cross‐sectional areas were measured on each axial slice, with their respective total volumes computed as the sum across slices within the abdominal compartment, leading to LV, SV, abdominal VAT volume and abdominal SAT volume. Liver and spleen mean attenuation values (LMA and SMA, respectively) were determined from the imaging scans to calculate SHAD, spleen attenuation minus liver attenuation. A greater or more positive value for SHAD corresponds with increased liver adiposity. We delineate HS through either a LMA < 40 HU or a SHAD ≥ −1 HU. This value for SHAD has previously been found to represent the cutoff for mild HS, as the typical SHAD cutoff of 10 HU is actually more concordant with the cutoff for moderate–severe HS.^21^, ^31^

### 2.4 Statistical analysis

All statistical tests were done using R (R Core Team, version 4.3.1; Foundation for Statistical Computing, Vienna, Austria). Patients were considered as diabetic if their HbA1c level was greater than or equal to 6.5%, while those that fell below this threshold were considered nondiabetic. Demographic information was collected. Moreover, IDPs from abdominal CT, such as LMA, SMA, SHAD, VAT, SAT and VSR, were determined. Hepatic steatosis prevalence in each cohort was calculated. Differences in categorical variables (sex and race) between cohorts were determined using the chi‐square test of independence. Differences in continuous variables (age, BMI, LMA, SHAD, VAT, SAT, VSR, HS prevalence) were determined using a two‐sided Wilcoxon rank‐sum test. Boxplots were created to compare IDP values with diabetes presence, stratified by sex. Multivariate regression analyses were performed for the presence of type 2 diabetes, looking independently at IDPs (SHAD, LV, SV, VAT, SAT and VSR). The model shown below (Model 1) represents the six iterations of the regression analyses performed with each IDP, controlling for sex, age, BMI and race. Odds ratios (ORs) were calculated from the multivariate analyses, including the 95% confidence interval (CI). *p*‐Values were adjusted with Benjamini–Hochberg false discovery rate (BH‐FDR) correction to reduce type 1 error with the statistical significance threshold of *p* < 0.05.

$$\text{Model}\;1:\log\;\frac{p\left(\mathrm{DIS}=1\right)}{p\left(\mathrm{DIS}=0\right)}=\mathrm{SEX}+\mathrm{AGE}+\mathrm{BMI}+\text{RACE}+\mathrm{IDP}.$$

Additionally, regression analysis was performed using a second model (Model 2), which combined the previously described IDPs into a single multivariable model that can assess whether individual phenotypes were independently associated with diabetes after accounting for correlations among imaging measures. To allow for comparison of effect sizes across the IDPs with different units, continuous variables were standardised to a mean of zero and a standard deviation of one for analysis in the combined multivariable model. In this model, standardised OR represents the change in odds of diabetes presence per one standard deviation increase in each continuous variable. VSR was used in the model in place of abdominal VAT and SAT to reduce multicollinearity while preserving information on the association between fat distribution and diabetes presence.

$$\text{Model}\;2:\log\frac{p\left(\mathrm{DIS}=1\right)}{p\left(\mathrm{DIS}=0\right)}=\begin{array}{l} \mathrm{SEX}+\mathrm{AGE}+\mathrm{BMI}+\text{RACE}+\text{SHAD}\\ +\;\mathrm{SV}+\mathrm{LV}+\mathrm{VSR}. \end{array}$$

## 3 RESULTS

### 3.1 Patient cohort

Patients were dichotomised by the HbA1c threshold of 6.5% (Table 1). No statistical significance was found between the nondiabetic and diabetic cohorts for age (62 vs. 62 years, *p* = 0.99) or sex (48.5% male vs. 50.8% male, *p* = 0.38). The diabetic cohort exhibited greater racial diversity; the percentage of Black patients was greater for the type 2 diabetes group than the nondiabetic counterpart (41.3% vs. 28.2%, *p* = 4.02 × 10^−7^), while the percentage of White patients was lower for the type 2 diabetes group (50.8% vs. 65.0%). Patients with type 2 diabetes had a higher BMI than patients without diabetes (31.2 vs. 29.3 kg/m^2^, *p* = 3.15 × 10^−7^). Patients with type 2 diabetes also had a higher median HbA1c value than patients without diabetes (5.7% vs. 7.6%, *p* < 2.2 × 10^−16^). Additionally, proportions of patients using select medication classes were found to be significantly higher in diabetic patients: metformin (43.8% vs. 15.2%, *p* < 2.2 × 10^−16^); sulfonylureas (20.8% vs. 4.6%, *p* < 2.2 × 10^−16^); glucagon‐like peptide‐1 agonists (11.3% vs. 3.1%, *p* = 6.75 × 10^−11^); sodium‐glucose cotransporter 2 inhibitors (5.4% vs. 0.7%, *p* = 2.31 × 10^−8^); and thiazolidinediones (3.4% vs. 1.4%, *p* = 1.03 × 10^−2^).

**TABLE 1:** Patient cohort demographics.

Total (*n* = 1594)	Nondiabetic (*n* = 950)	T2DM (*n* = 644)	*p*‐Value
Age (years)	62 [52, 70]	62 [53, 69]	0.99
Sex (*n* [%])			0.38
Male	461 (48.5)	327 (50.8)	
Female	489 (51.5)	317 (49.2)	
Race (*n* [%])			4.02 × 10^−7^
White	618 (65.0)	327 (50.8)	
Black	268 (28.2)	266 (41.3)	
Asian/PI	18 (1.9)	20 (3.1)	
Hispanic/Latino	22 (2.3)	16 (2.5)	
Other	24 (2.5)	15 (2.3)	
BMI (kg/m^2^)	29.3 [25.4, 34.7]	31.2 [27.2, 36.5]	3.15 × 10^−7^
HbA1c (%)	5.7 [5.4, 6.0]	7.6 [6.9, 9.1]	<2.2 × 10^−16^
Medications (*n* [%])			
Metformin	144 (15.2)	282 (43.8)	<2.2 × 10^−16^
Sulfonylureas	44 (4.6)	134 (20.8)	<2.2 × 10^−16^
GLP‐1 agonists	29 (3.1)	73 (11.3)	6.75 × 10^−11^
SGLT2 inhibitors	7 (0.7)	35 (5.4)	2.31 × 10^−8^
TZDs	13 (1.4)	22 (3.4)	1.03 × 10^−2^

*Note*: The demographic characteristics of included patients from the Penn Medicine BioBank. Age, sex, HbA1c are arranged as median [Q1, Q3]. Sex and race are organised as sample size (percentage). *p*‐Values for continuous variables (age, BMI) were calculated using a Wilcoxon rank‐sum test and for categorical variables (sex, race) using a chi‐square test of independence to determine statistically significant differences between the ‘Nondiabetic’ and ‘T2DM’ groups.

Abbreviations: BMI, body mass index; GLP‐1, glucagon‐like peptide‐1; HbA1c, haemoglobin A1c; PI, Pacific Islander; SGLT2, sodium‐glucose cotransporter 2; T2DM, type 2 diabetes mellitus; TZD, thiazolidinediones.

Hepatic and abdominal IDPs were quantified and stratified based on type 2 diabetes diagnosis (Table 2). Both SV (0.19 L vs. 0.18 L, *p* = 2.67 × 10^−2^) and LV (1.74 L vs. 1.58 L, *p* = 5.02 × 10^−8^) were larger in the type 2 diabetes patient cohort than in the nondiabetes cohort. LMA for diabetics was lower than for nondiabetics (47.0 vs. 50.9 HU, *p* = 1.36 × 10^−10^), as well as SMA (43.2 vs. 44.3 HU, *p* = 2.67 × 10^−2^). SHAD was greater in diabetics (−4.49 vs. –6.88 HU, *p* = 1.34 × 10^−8^). The presence of HS was greater in the diabetic cohort (41.8% vs. 27.7%, *p* = 4.85 × 10^−9^). The VAT volume (3.45 vs. 2.53 L, *p* = 3.12 × 10^−12^) and VAT area (190.82 vs. 148.71 cm^2^, *p* = 1.01 × 10^−10^), as well as SAT volume (5.43 vs. 4.47 L, *p* = 7.39 × 10^−7^) and SAT area (297.76 vs. 258.06 cm^2^, *p* = 2.83 × 10^−5^) were greater in diabetic patients. VSR was higher in patients with type 2 diabetes (0.62 vs. 0.55, *p* = 1.69 × 10^−3^).

**TABLE 2:** Imaging derived phenotype (IDP) values using computed tomography from the patient cohort.

Total (*n* = 1594)	Nondiabetic (*n* = 950)	T2DM (*n* = 644)	*p*‐Value
Hepatic IDP values			
LMA (HU)	50.9 [44.0, 56.6]	47.0 [39.3, 54.3]	1.36 × 10^−10^
SMA (HU)	44.3 [39.1, 48.0]	43.2 [37.6, 47.8]	2.67 × 10^−2^
SHAD (HU)	−6.88 [−12.0, −1.47]	−4.49 [−10.1, 2.53]	1.34 × 10^−8^
HS prevalence (*n* [%])	263 (27.7)	269 (41.8)	4.85 × 10^−9^
LV (L)	1.58 [1.30, 1.94]	1.74 [1.41, 2.12]	5.02 × 10^−8^
SV (L)	0.18 [0.12, 0.26]	0.19 [0.12, 0.28]	2.67 × 10^−2^
Abdominal fat IDP values			
VAT volume (L)	2.53 [1.33, 4.06]	3.45 [2.04, 5.02]	3.12 × 10^−12^
SAT volume (L)	4.47 [2.78, 7.15]	5.43 [3.54, 7.83]	7.39 × 10^−7^
VAT area (cm^2^)	148.71 [91.26, 227.26]	190.82 [124.16, 272.0]	1.01 × 10^−10^
SAT area (cm^2^)	258.06 [175.08, 390.40]	297.76 [207.92, 428.26]	2.83 × 10^−5^
VSR	0.55 [0.34, 0.89]	0.62 [0.39, 0.97]	1.69 × 10^−3^

*Note*: LV, SV, SMA, LMA, SHAD, VAT, SAT and VSR are arranged as median [Q1, Q3]. HS prevalence for each group is formatted as sample size (percentage), where SHAD ≥−1 HU or LMA < 40 HU is classified as a HS diagnosis. *p*‐Values were calculated using a Wilcoxon rank‐sum test to determine statistically significant differences between the ‘Nondiabetic’ and ‘T2DM’ groups and were corrected using Benjamini–Hochberg false discovery rate correction for multiple testing.

Abbreviations: HS, hepatic steatosis; HU, Hounsfield unit; LMA, liver mean attenuation value; LV, liver volume; SAT, subcutaneous adipose tissue; SHAD, spleen‐hepatic attenuation difference; SMA, spleen mean attenuation value; SV, spleen volume; T2DM, type 2 diabetes mellitus; VAT, visceral adipose tissue; VSR, visceral‐to‐subcutaneous fat volume ratio.

These trends generally held when stratifying by sex (Figure S3), as women with diabetes showed significantly higher SHAD (−4.99 vs. –7.97 HU, *p* = 8.62 × 10^−5^), VSR (0.43 vs. 0.36, *p* = 3.41 × 10^−4^), VAT (2.66 vs. 2.01 L, *p* = 1.92 × 10^−8^), SAT (6.23 vs. 5.16 L, *p* = 9.14 × 10^−4^) and LV (1.57 vs. 1.42 L, *p* = 2.44 × 10^−6^) than women without diabetes, and men with diabetes showed significantly higher SHAD (−4.15 vs. –5.64 HU, *p* = 1.61 × 10^−4^), VAT (4.37 vs. 3.46 L, *p* = 8.32 × 10^−7^), SAT (4.98 vs. 3.81 L, *p* = 2.63 × 10^−5^) and LV (1.87 vs. 1.74 L, *p* = 1.92 × 10^−3^) than men without diabetes. However, VSR values between diabetic and nondiabetic males, as well as SV values between diabetic and nondiabetic males and females were nonsignificant. Between the sexes, median SHAD (−4.15 vs. −4.99 HU, *p* = 4.05 × 10^−1^), VSR (0.93 vs. 0.43, *p* < 2.2 × 10^−16^), VAT (4.37 vs. 2.66 L, *p* < 2.2 × 10^−16^), LV (1.87 vs. 1.57 L, *p* = 3.84 × 10^−9^) and SV (0.23 vs. 0.16 L, *p* = 3.82 × 10^−11^) were significantly higher among male diabetics, while abdominal SAT (6.23 vs. 4.98 L, *p* = 9.55 × 10^−8^) volume was significantly higher in female diabetics.

### 3.2 Multivariate analysis

The multivariate analyses examined ORs of hepatic and abdominal IDPs with type 2 diabetes presence, controlling for age, sex, race and BMI (Table 3). When examining the IDPs specifically, all except abdominal SAT were independently associated with diabetes: SHAD (OR 1.04, 95% CI [1.02–1.05]); LV (OR 1.63, 95% CI [1.31–2.04]); SV (OR 4.53, 95% CI [1.89–10.99]); VAT (OR 1.24, 95% CI [1.17–1.33]); and VSR (OR 2.87, 95% CI [1.96–4.20]).

**TABLE 3:** Odds ratio model of type 2 diabetes presence against abdominal adipose imaging phenotype values and demographic characteristics.

Variables	Specific IDP variables											
	IDP: SHAD		IDP: LV		IDP: SV		IDP: VAT		IDP: SAT		IDP: VSR	
	OR (95% CI)	*p*‐Value	OR (95% CI)	*p*‐Value	OR (95% CI)	*p*‐Value	OR (95% CI)	*p*‐Value	OR (95% CI)	*p*‐Value	OR (95% CI)	*p*‐Value
Age	1.01 (1.00–1.02)	7.21 × 10^−2^	1.01 (1.00–1.02)	2.59 × 10^−2^	1.01 (1.00–1.02)	2.82 × 10^−2^	1.00 (0.99–1.01)	0.72	1.01 (1.00–1.02)	0.13	1.00 (0.99–1.01)	0.80
Sex^a^												
Male	1.24 (1.00–1.53)	7.21 × 10^−2^	1.09 (0.87–1.37)	0.52	1.18 (0.95–1.47)	0.18	0.87 (0.68–1.11)	0.43	1.31 (1.06–1.63)	3.47 × 10^−2^	0.81 (0.62–1.07)	0.20
Race/ethnicity^a^												
Black	1.97 (1.56–2.48)	3.76 × 10^−8^	1.97 (1.57–2.49)	6.58 × 10^−8^	2.11 (1.66–2.69)	1.84 × 10^−8^	2.28 (1.80–2.90)	6.18 × 10^−11^	1.82 (1.45–2.29)	1.77 × 10^−6^	2.26 (1.77–2.88)	3.46 × 10^−10^
Asian/PI	2.57 (1.31–5.07)	1.21 × 10^−2^	2.96 (1.51–5.82)	4.01 × 10^−3^	2.79 (1.43–5.49)	5.14 × 10^−3^	2.51 (1.28–4.94)	1.92 × 10^−2^	2.48 (1.18–4.86)	2.83 × 10^−2^	2.49 (1.27–4.90)	1.52 × 10^−2^
Hispanic/Latino	1.60 (0.80–3.13)	0.20	1.34 (0.58–3.24)	0.31	1.41 (0.61–3.39)	0.18	1.46 (0.63–3.55)	0.31	1.32 (0.57–3.16)	0.32	1.46 (0.63–3.52)	0.20
Other	1.37 (0.69–2.66)	0.35	1.22 (0.61–2.35)	0.56	1.22 (0.61–2.35)	0.57	1.24 (0.62–2.42)	0.72	1.18 (0.60–2.27)	0.62	1.28 (0.64–2.48)	0.55
BMI	1.03 (1.01–1.04)	8.78 × 10^−4^	1.02 (1.00–1.03)	7.66 × 10^−2^	1.03 (1.01–1.04)	2.40 × 10^−3^	1.00 (0.98–1.01)	0.72	1.02 (1.00–1.05)	5.69 × 10^−2^	1.03 (1.02–1.05)	2.29 × 10^−5^
IDP (see column headers)	1.04 (1.02–1.05)	8.72 × 10^−9^	1.63 (1.31–2.04)	6.55 × 10^−5^	4.53 (1.89–10.99)	2.40 × 10^−3^	1.24 (1.17–1.33)	6.18 × 10^−11^	1.02 (0.97–1.07)	0.51	2.87 (1.96–4.20)	2.32 × 10^−7^

*Note*: Six iterations of logistic regressions (derived from Model 1) were performed, with different IDPs each time that are listed in the column headers. *p*‐Values were adjusted with Benjamini–Hochberg (false discovery rate) correction with the statistical significance threshold *p* < 0.05.

Abbreviations: BMI, body mass index; CI, confidence interval; IDP, image‐derived phenotype; LV, liver volume; SAT, abdominal subcutaneous adipose tissue volume; OR, odds ratios; PI, Pacific Islander; SHAD, spleen‐hepatic attenuation difference; SV, spleen volume; VAT, abdominal visceral adipose tissue volume; VSR, visceral‐to‐subcutaneous fat ratio.

^a^ The baseline category for the sex variable was female and for the race variable was White.

The analysis using the combined model for multiple abdominal IDPs (Figure S4) yielded independent associations for SHAD (OR 1.03, 95% CI [1.02–1.04]), LV (OR 1.36, 95% CI [1.07–1.74]) and VSR (OR 2.36, 95% CI [1.60–3.49]). SV was no longer independently associated with type 2 diabetes presence after controlling for hepatic and abdominal fat distribution IDPs (OR 1.86, 95% CI [0.70–4.90]). When the continuous predictors in the model were standardised, VSR (OR 1.40, 95% CI [1.20–1.63]) exhibited a larger association with diabetes presence than BMI, LV, SV and SHAD.

Even after removing BMI from the multivariate analyses (Figure S5), all of the IDPs were associated with diabetes: SHAD (OR 1.04, 95% CI [1.03–1.05]); LV (OR 1.80, 95% CI [1.47–2.20]); SV (OR 6.36, 95% CI [2.71–15.12]); VAT (OR 1.24, 95% CI [1.17–1.30]); SAT (OR 1.06, 95% CI [1.03–1.10]); and VSR (OR 2.73, 95% CI [1.87–3.99]).

## 4 DISCUSSION

In this study, we applied an automated machine learning algorithm to imaging and clinical data from a tertiary‐care academic biobank to characterise hepatic and abdominal adiposity patterns in patients with and without type 2 diabetes. Our results demonstrate robust associations on CT scans between various metrics associated with abdominal obesity‐related features, including HS, hepatosplenomegaly and the overall distribution of abdominal adipose tissue, with the presence of type 2 diabetes. Prior studies have examined the relationship of individual image‐derived abdominal adipose values with type 2 diabetes, which limits the ability to compare the relative contribution of different fat compartments or organ characteristics to disease presence.^14^, ^15^, ^16^, ^18^ Our study uses an integrative approach to simultaneously evaluate these abdominal IDPs on a large‐scale basis, which enables a comprehensive assessment of metabolic imaging features and their relative contributions to the presence of type 2 diabetes.

Among abdominal adiposity values, abdominal VAT and VSR have the strongest associations with type 2 diabetes presence, holding BMI constant. Because BMI does not delineate body fat distribution and muscle mass,^32^, ^33^ VSR and VAT may serve as alternative metrics for obesity and better reflect the relationship of abdominal obesity to type 2 diabetes.^34^, ^35^, ^36^, ^37^ VSR exhibited a stronger association with type 2 diabetes presence than BMI in the standardised combined regression, which supports the notion that fat distribution conveys information beyond overall adiposity.^38^, ^39^ Even after removing BMI from the regression, VSR still has an independent association with diabetes, suggesting that VSR captures a unique aspect of type 2 diabetes.^40^ In contrast, abdominal SAT volume was not associated with diabetes presence in models with BMI adjustment; however, the association was observed after BMI was excluded from the model, likely reflecting collinearity between BMI and abdominal SAT volume. Previous studies have found that SAT's relationship with diabetes is nuanced, with some evidence indicating that the specific location of SAT may even provide some protective effect against development of the disease.^36^, ^41^, ^42^ Further research is necessary to comprehend the complex relationship between abdominal SAT and diabetes.

Hepatic steatosis, quantified through SHAD, was also strongly associated with diabetes presence in our cohort.^43^ While a SHAD of 10 HU is widely used by radiologists as the cutoff for moderate‐to‐severe HS, −1 HU has been histologically proven to coincide with at least mild steatotic liver disease.^31^ By redefining the parameters of HS to make it more sensitive, we captured a larger proportion of patients with more mild degrees of HS and showed its association with type 2 diabetes. Moreover, the observed associations between diabetes and increased liver and SVs highlight how metabolic dysfunction and fatty infiltration can manifest as detectable structural organ changes on imaging.^16^, ^18^ The relationship between SV and type 2 diabetes has been relatively understudied^18^, ^19^; here we demonstrate that SV is independently correlated with type 2 diabetes presence on CT, but the effect does not persist after controlling for hepatic and abdominal adiposity. Prior MRI‐based studies have found larger SVs in type 2 diabetes patients, but SV is not independently associated with the disease after controlling for liver‐related factors, supporting the interpretation that splenic changes are secondary to hepatic manifestations rather than directly interconnected to type 2 diabetes.^44^, ^45^

Beyond these associations, the methodology used in this study has important implications for clinical research. Opportunistic and automated analysis of CT imaging allows for the large‐scale extraction of metabolic risk factors without presenting additional cost, time for manual quantification or patient burden.^8^, ^12^ Prior CT‐based studies have quantified various data such as bone mineral density, aortic calcium and skeletal muscle mass to predict metabolic syndromes and cardiovascular disease.^46^, ^47^, ^48^ By combining EHR clinical data, such IDPs may support population‐level type 2 diabetes risk stratification and enable future studies to predict disease progression or incident diabetes.^47^, ^49^, ^50^

This study has several strengths, such as the racial diversity of the PMBB patient population, although some racial cohorts had small sample sizes (such as Asian/Pacific Islander and Hispanic/Latino). Also, the biobank data is taken from patients who have diseases, whereas the UK Biobank consists mainly of healthy participants.^51^ While the biobank's patient data is skewed to the geographic and demographic characteristics of southeastern Pennsylvania and New Jersey,^52^ it does include patients from multiple hospitals and clinics in urban, suburban and rural settings. A limitation of this study was the use of one CT scan to be representative of the patient. Although we could define associations between adiposity and diabetes, given the study's cross‐sectional design, we were unable to determine a causal relationship of these IDPs to the onset of diabetes or track disease pathogenesis across CT scan dates. Lastly, the use of phecodes to infer certain conditions presented some challenges since phecodes can imperfectly distinguish or group certain phenotypes.^53^ The use of HbA1c values within 1 year of the abdominal CT scan to define diabetes status, rather than phecodes, improves temporal relevance as phecodes may reflect historical diagnoses or incomplete coding at the date of CT acquisition. Multi‐centre observational longitudinal studies that examine abdominal IDPs over consecutive abdominal CT scans can provide insight into how hepatic and abdominal fat distribution changes influence diabetes progression.

In future research, we hope to further elucidate the relationship between diabetes incidence, HS and abdominal fat distribution. A Korean study determined the VSR cutoff for men and women for predicting the incidence of type 2 diabetes, but no study has been done yet in America, which is more heterogeneous.^35^, ^40^ VSR holds promise as a metric to more accurately describe abdominal obesity and adiposity, and we hope to see it calculated and employed within clinical practice soon. Moreover, incorporation of the nutritional data of this patient population can provide insight into how diet and dietary modifications influence abdominal adiposity and disease aetiology.^54^ Current research is underway to explore how diet can cause changes in imaging before clinical manifestations occur.^55^

In summary, HS, obesity and diabetes are interlinked findings, and their mild presentations potentially can serve as harbingers for more severe, even life‐threatening disease.^1^, ^3^, ^38^ By applying artificial intelligence and using EHR data to characterise the relationship of IDPs with diabetes, we have confirmed and established novel associations that serve as a high‐level overview to encourage future inquiry into how adiposity is related to diabetes mellitus. This study presents a scalable framework for the use of machine learning to capture IDPs in a diabetic population to catalyse translational science.

## AUTHOR CONTRIBUTIONS

**RHT**: Conceptualisation, methodology, validation, formal analysis, investigation, data curation, writing—original draft, writing—review and editing, visualisation. **PR**: Conceptualisation, methodology, software, validation, formal analysis, investigation, data curation, writing—review and editing, visualisation. **MH**: Conceptualisation, methodology, formal analysis, investigation, data curation. **ET**: Conceptualisation, methodology, software, formal analysis, investigation, data curation. **SS**: Methodology, validation, investigation. **ABh**: Methodology, validation, investigation. **MM**: Methodology, software, validation, formal analysis, investigation, data curation. **JTD**: Methodology, software, validation, formal analysis, investigation, data curation. **JG**: Methodology, resources, data curation. **CK**: Methodology, validation, investigation. **DJR**: Resources, data curation. **ABo**: Methodology, validation, investigation. **WRW**: Conceptualisation, methodology, investigation, resources, data curation, writing—review and editing, supervision. **HS**: Conceptualisation, methodology, investigation, resources, data curation, writing—review and editing, visualisation, supervision.

## FUNDING INFORMATION

Walter R. Witschey is supported by National Institutes of Health (NIH) National Heart, Lung, and Blood Institute (NHLBI) grants R01HL171709 and R01HL169378 and NIH National Institute of Biomedical Imaging and Bioengineering (NIBIB) grant P41EB029460. Hersh Sagreiya was supported by a Radiological Society of North America Research Scholar Grant #RSCH2028 and was supported in part by the Institute for Translational Medicine and Therapeutics' (ITMAT) Transdisciplinary Program in Translational Medicine and Therapeutics. Walter R. Witschey, Hersh Sagreiya, James Gee, and Jeffrey T. Duda were supported by the NIH Office of the Director grant OT2OD038048. Walter R. Witschey and Hersh Sagreiya were supported by NIH NIBIB grant R21EB036734. The PMBB is supported by the Perelman School of Medicine at the University of Pennsylvania, a gift from the Smilow family, and the National Center for Advancing Translational Sciences of the National Institutes of Health under CTSA award number UL1TR001878. The content is solely the responsibility of the authors and does not represent the views of the Department of Veterans Affairs, the National Institutes of Health, or the United States Government. Matthew MacLean received funding from the Sarnoff Cardiovascular Research Foundation.

## CONFLICT OF INTEREST STATEMENT

The authors declare no competing interests.

## Supporting information


**Data S1.** Supporting Information.

## Data Availability

The data that support the findings of this study are available from the corresponding author upon reasonable request.
