# Supplementary material for: Hepatic and abdominal adiposity in type 2 diabetes as assessed with machine learning on computed tomography scans
Source: Diabetes Obes Metab. 2026 Feb 18;28(5):3742–51. doi: 10.1111/dom.70557 (PMC13071195; doi:10.1111/dom.70557)
Supplement: Supplementary file 1 — Data S1. Supporting Information. [file DOM-28-3742-s001.docx]

Supplementary Figure 1: CPT codes

| CPT Code | Description |
| --- | --- |
| 74176 | CT abdomen and pelvis without contrast |
| 74178 | CT abdomen and pelvis with and without contrast |
| 74150 | CT abdomen without contrast |
| 74170 | CT abdomen with and without contrast |
| 71250 | non-contrast chest CT (diagnostic) |
| 71270 | non-contrast chest CT, with follow up CT scans (diagnostic) |

CPT codes used to identify computed tomography scans that had views of the abdomen. CPT = Current Procedural Terminology.

Supplementary Figure 2: ICD-9 codes and associated phecodes

| Phecode | ICD-9 Mapped Codes |
| --- | --- |
| Alcohol Use Disorders (317, 317.1, 317.11) | 291, 291.0-291.9, 303, 305.0, 305.00-305.03, 357.5, 535.3, 571.0-571.3, 790.3, 980.0 |
| Liver abscess and sequelae of chronic liver disease (571.8) | 572, 572.0-572.8 |
| Hepatitis (070, 070.1-070.4, 070.9) | 070. 573.1, 573.2, 571.3, 571.4 |

Phecodes are listed as phecode description (phecode number). Phecodes are mapped from ICD-9 (<https://phewascatalog.org/phecodes>). ICD-9 = International Classification of Diseases, Ninth Revision.

Supplementary Figure 3: Box-and-whisker plots of IDPs in diabetic and nondiabetic patient cohorts categorized by sex

Boxplots show the distribution of image-derived phenotypes (IDPs) including liver–spleen attenuation difference (SHAD), visceral–subcutaneous fat ratio (VSR), visceral adipose tissue (VAT), subcutaneous adipose tissue (SAT), liver volume (LV), and spleen volume (SV) across four groups: Female/ND (non-diabetic), Female/T2D (type 2 diabetes), Male/ND, and Male/T2D. Median values are displayed above while IQR values are displayed below. Pairwise differences between groups were assessed using the two-sided Wilcoxon rank-sum test with Benjamini–Hochberg false discovery rate (BH-FDR) correction for multiple testing. Only significant pairwise comparisons are shown. Statistical significance is denoted by asterisks as follows: * *p* < 0.05; ** *p* < 0.01; *** *p* < 0.001; **** *p* < 0.0001.

Supplementary Figure 4: Odds ratio model of type 2 diabetes presence against cumulative abdominal adipose imaging phenotype values and demographic characteristics

| Variable | OR (95% CI) | Scaled OR (95% CI) | P-value |
| --- | --- | --- | --- |
| Age | 1.01 (1.00-1.02) | 1.09 (0.97-1.22) | 0.18 |
| Sex |  |  |  |
| Male | 0.73 (0.55-0.98) | 0.73 (0.55-0.98) | 5.30x10^-2^ |
| Race |  |  |  |
| Black | 2.56 (1.98-3.32) | 2.56 (1.98-3.32) | 9.59x10^-12^ |
| Asian/PI | 2.98 (1.49-5.98) | 2.98 (1.49-5.98) | 5.24x10^-3^ |
| Hispanic/Latino | 1.77 (0.88-3.50) | 1.77 (0.88-3.50) | 0.14 |
| Other | 1.48 (0.74-2.89) | 1.48 (0.74-2.89) | 0.26 |
| BMI | 1.02 (1.00-1.04) | 1.14 (1.01-1.29) | 5.30x10^-2^ |
| Abdominal IDPs |  |  |  |
| SHAD | 1.03 (1.02-1.04) | 1.31 (1.18-1.46) | 3.90x10^-6^ |
| LV | 1.36 (1.07-1.74) | 1.19 (1.04-1.36) | 2.70x10^-2^ |
| SV | 1.86 (0.70-4.90) | 1.08 (0.95-1.23) | 0.23 |
| VSR | 2.36 (1.60-3.49) | 1.40 (1.20-1.63) | 5.94x10^-5^ |

Six iterations of logistic regressions (derived from Model 1) were performed, with different IDPs each time that are listed in the column headers. P-values were adjusted with Benjamini-Hochberg (FDR) correction with the statistical significance threshold p<0.05. *The baseline category for the sex variable was female and for the race variable was White. BMI = body mass index, IDP = image-derived phenotype, SHAD = spleen hepatic attenuation difference, LV = liver volume, SV = spleen volume, VAT = abdominal visceral adipose tissue volume, SAT = abdominal subcutaneous adipose tissue volume, VSR = visceral-to-subcutaneous fat ratio.

Supplementary Figure 5: Odds ratio model of type 2 diabetes presence against abdominal adipose imaging phenotype values and demographic characteristics without BMI

|  | Specific IDP Variables | | | | | | | | | | | |
| --- | --- | --- | --- | --- | --- | --- | --- | --- | --- | --- | --- | --- |
| Variables | IDP: SHAD | | IDP: LV | | IDP: SV | | IDP: VAT | | IDP: SAT | | IDP: VSR | |
|  | OR (95% CI) | p-value | OR (95% CI) | p-value | OR (95% CI) | p-value | OR (95% CI) | p-value | OR (95% CI) | p-value | OR (95% CI) | p-value |
| **Age** | 1.01 (1.00-1.01) | 0.20 | 1.01 (1.00-1.02) | 3.21x10^-2^ | 1.01 (1.00-1.02) | 7.75x10^-2^ | 1.00 (0.99-1.01) | 0.57 | 1.01 (1.00-1.01) | 0.15 | 1.00 (0.99-1.01) | 0.68 |
| **Sex*** |  | | | | | | | | | | | |
| Male | 1.18 (0.96-1.47) | 0.20 | 1.03 (0.83-1.29) | 0.77 | 1.12 (0.90-1.39) | 0.36 | 0.88 (0.70-1.11) | 0.41 | 1.32 (1.07-1.64) | 2.11x10^-2^ | 0.79 (0.60-1.04) | 0.16 |
| **Race*** |  | | | | | | | | | | | |
| Black | 2.12 (1.69-2.66) | 3.75x10^-10^ | 2.07 (1.66-2.60) | 1.77x10^=9^ | 2.32 (1.83-2.94) | 2.05x10^-11^ | 2.25 (1.79-2.84) | 1.62x10^-11^ | 1.85 (1.48-2.33) | 6.46x10^-7^ | 2.44 (1.93-3.11) | 1.61x10^-12^ |
| Asian/PI | 2.25 (1.16-4.42) | 3.88x10^-2^ | 2.87 (1.47-5.64) | 4.59x10^-3^ | 2.55 (1.32-4.99) | 1.29x10^-2^ | 2.53 (1.30-4.98) | 1.48x10^-2^ | 2.32 (1.20-4.53) | 2.11x10^–2^ | 2.12 (1.10-4.15) | 5.94x10^-2^ |
| Hispanic/ Latino | 1.61 (0.81-3.14) | 0.20 | 1.52 (0.76-2.97) | 0.32 | 1.65 (0.83-3.22) | 0.20 | 1.60 (0.80-3.14) | 0.30 | 1.49 (0.75-2.89) | 0.29 | 1.64 (0.83-3.20) | 0.20 |
| Other | 1.39 (0.70-2.68) | 0.34 | 1.23 (0.62-2.37) | 0.64 | 1.23 (0.62-2.36) | 0.55 | 1.24 (0.61-2.41) | 0.57 | 1.18 (0.60-2.27) | 0.62 | 1.28 (0.64-2.48) | 0.55 |
| **IDP (see column headers)** | 1.04 (1.03-1.05) | 3.75x10^-10^ | 1.80 (1.47-2.20) | 6.10x10^-8^ | 6.36 (2.71-  15.12) | 8.57x10^-5^ | 1.24 (1.17-1.30) | 1.49x10^-14^ | 1.06 (1.03-1.10) | 8.48x10^-4^ | 2.73 (1.87-3.99) | 6.56x10^-7^ |

Six iterations of logistic regressions (derived from Model 1 without BMI) were performed, with different IDPs each time that are listed in the column headers. P-values were adjusted with Benjamini-Hochberg correction with the statistical significance threshold of p<0.05. *The baseline category for the sex variable was female and for race variable was White. IDP = image-derived phenotype, SHAD = spleen hepatic attenuation difference, LV = liver volume, SV = spleen volume, VAT = visceral adipose tissue volume, SAT = subcutaneous adipose tissue volume, VSR = visceral-to-subcutaneous fat ratio.
